# Peripheral immune biomarkers for immune checkpoint inhibition of solid tumours

**DOI:** 10.1002/ctm2.1814

**Published:** 2024-08-20

**Authors:** Meghali Goswami, Nicole J. Toney, Stephanie C. Pitts, Carolina Celades, Jeffrey Schlom, Renee N. Donahue

**Affiliations:** ^1^ Center for Immuno‐Oncology, Center for Cancer Research, National Cancer Institute National Institutes of Health Bethesda Maryland USA

**Keywords:** biomarkers, immunotherapy, peripheral blood, tumour microenvironment

## Abstract

**Background:**

With the rapid adoption of immunotherapy for the treatment of cancer comes the pressing need for readily accessible biomarkers to guide immunotherapeutic strategies and offer insights into outcomes with specific treatments. Regular sampling of solid tumour tissues outside of melanoma for immune monitoring is not often feasible; conversely, routine, frequent interrogation of circulating immune biomarkers is entirely possible. As immunotherapies and immune checkpoint inhibitors, in particular, are more widely used in first‐line, neoadjuvant, and metastatic settings, the discovery and validation of peripheral immune biomarkers are urgently needed across solid tumour types for improved prediction and prognostication of clinical outcomes in response to immunotherapy, as well as elucidation of mechanistic underpinnings of the intervention. Careful experimental design, encompassing both retrospective and prospective studies, is required in such biomarker identification studies, and concerted efforts are essential for their advancement into clinical settings.

**Conclusion:**

In this review, we summarize shared immune features between the tumour microenvironment and systemic circulation, evaluate exploratory peripheral immune biomarker studies, and discuss associations between candidate biomarkers with clinical outcomes. We also consider integration of multiple peripheral immune parameters for better prediction and prognostication and discuss considerations in study design to further evaluate the clinical utility of candidate peripheral immune biomarkers for immunotherapy of solid tumours.

**Highlights:**

- Peripheral immune biomarkers are critical for improved prediction and prognostication of clinical outcomes for patients with solid tumours treated with immune checkpoint inhibition.
- Candidate peripheral biomarkers, such as cytokines, soluble factors, and immune cells, have potential as biomarkers to guide immunotherapy of solid tumours.
- Multiple peripheral immune parameters may be integrated to improve prediction and prognostication.
- The potential of peripheral immune biomarkers to guide immunotherapy of solid tumours requires critical work in biomarker discovery, validation, and standardization.

## Graphical abstract

- Discovery and validation of peripheral immune biomarkers are critical for improved prediction and prognostication of clinical outcomes for patients with solid tumours in response to immune checkpoint inhibition.
- Many candidate peripheral biomarkers, including cytokines, soluble factors, and immune cells have the potential as biomarkers to guide immunotherapy of solid tumours.
- Integration of multiple peripheral immune parameters may improve prediction and prognostication.
- Critical work in all phases of the biomarker discovery, validation, and standardization process is required to realize the potential of peripheral immune biomarkers to guide immunotherapy.

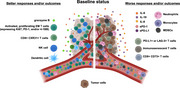

## 1 INTRODUCTION

Since the innovation of immune checkpoint inhibition (ICI), many immunotherapeutic modalities have been developed and moved into the clinic. Responses to immunotherapy are variable across tumour types, and biomarkers that identify patients likely to have anti‐tumour responses are crucial for their further advancement and utility.^1^ A biomarker, a portmanteau of “biological marker,” is an objective and measurable characteristic of a biological state. There are several categories of biomarkers: diagnostic (indicates whether an individual has a specific condition), prognostic (informs risk of a future outcome), and predictive (determines likelihood of response to a specific intervention).^2^ Biomarkers can belong to more than one category. Predictive and prognostic immune‐related biomarkers in the context of cancer can allow for refinement of patient selection, enable early estimation of the clinical efficacy of immunotherapies, and improve outcomes on a personalized patient basis. Current FDA‐approved predictive biomarkers for immunotherapy of solid tumours include tumour PD‐L1 protein expression, microsatellite instability (MSI), and tumour mutational burden (TMB). These biomarkers are measured with tumour tissue, though liquid biopsy TMB may be accepted in place of tumour TMB and MSI status (FoundationOne CDx).^3^

Immune‐related biomarkers are attractive prospects to guide immunotherapy of solid tumours, as immunotherapies potentiate the immune system in myriad ways. For example, the density and localization of tumour‐infiltrating lymphocytes (TILs) within the tumour core or along the margins are promising experimental tumour tissue‐derived biomarkers.^4^ However, routine assessment of TILs through repeated sampling of tumour tissue is highly invasive and painful for patients, making repeat biopsies largely unfeasible. It is considerably easier to perform multi‐omic analyses of peripheral blood and simultaneously interrogate many circulating correlatives. While immunological biomarkers measured from peripheral blood have not yet made their way into clinical settings, this is a vital area of research, as routine minimally invasive sampling of blood allows for tailoring of immunotherapeutic regimens to maximize anti‐tumour activity.

**FIGURE 1:**
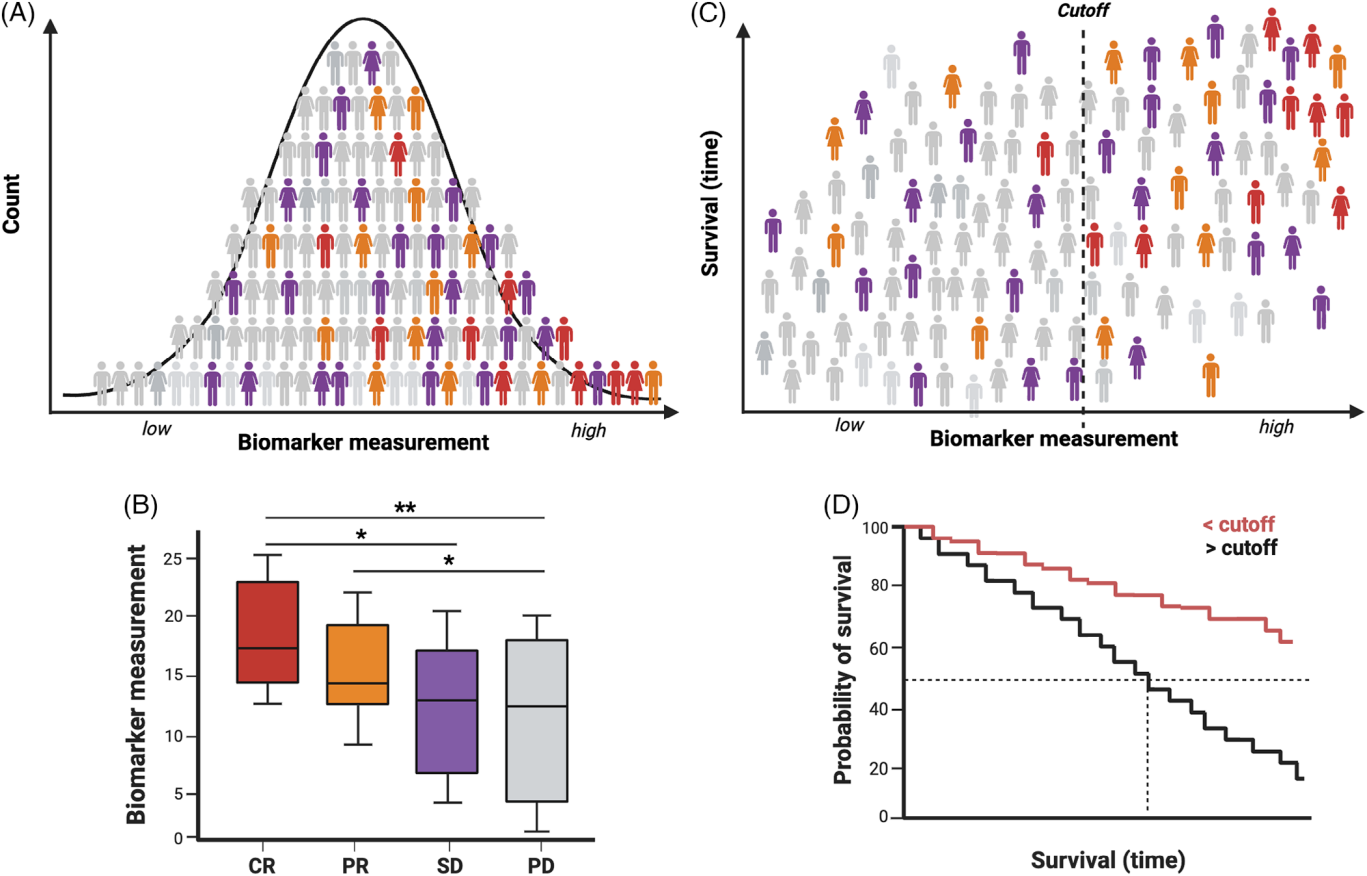
Evaluating associations between candidate biomarkers and clinical outcomes. (A) Phenotypic variation of a peripheral immune biomarker measured in a cohort receiving an immunotherapeutic intervention will fall along a distribution. (B) Predictive biomarker studies typically assess whether measurements of the candidate biomarker statistically vary in patients based on their anti‐tumour responses. (C) Survival metrics, including but not limited to progression‐free survival and overall survival, can be tabulated for the cohort, and the distribution can be dichotomized with cutoffs to determine associations between the biomarker with the survival metric. (D) Kaplan–Meier survival analyses are typically used to evaluate the time elapsed to an event (progression, death, etc.) in patients stratified into groups by the cutoff measurement of the biomarker. An association between a biomarker with response (i.e., predictive) may not necessarily mean that biomarker is associated with survival (i.e., prognostic). CR, complete response; PD, progressive disease; PR, partial response; SD, stable disease. Created with BioRender.com.

For a cancer biomarker to be clinically useful, it must pass many stages of development and validation to prove its utility and reliability. Initial identification of putative biomarkers typically derives from hypothesis‐generating studies. Most often, studies report correlations between biological characteristics (concentration of a given serum protein, frequency of a cell type, etc.) with a clinical outcome such as best overall response (BOR) or survival in a very specific setting (Figure 1). In this review, we focus on potentially predictive and prognostic immune‐related biomarkers from peripheral blood that may enable the guiding of ICI for solid tumours. Since melanoma biopsies in most cases are easily accessible, we focus on studies from other solid tumours. We survey the literature on candidate peripheral immune biomarkers and discuss associations with clinical response in a specific setting with specific immunotherapy and survival metrics.

## 2 SHARED FEATURES BETWEEN THE TUMOUR MICROENVIRONMENT AND THE PERIPHERAL IMMUNOME

Elucidation of immune cells and tertiary lymphoid structures populating tumours has advanced our understanding of intratumoural immune organization, and this contexture has implications for prognosis.^5^, ^6^ The tumour microenvironment (TME) is extensively linked to the systemic immune system, with complex vasculature bringing immune cells from the periphery into contact with the margins of the cancerous tissue.^7^ There is constant movement between the TME and systemic circulation and the potential of shared immune‐related features.

In patients with triple‐negative breast cancer, higher frequencies of tumour‐infiltrating CD4^+^ T cells expressing PD‐1 and CD39 and CD8^+^ T cells expressing CD39 corresponded to higher frequencies of these phenotypes in the periphery.^8^ In patients with pancreatic cancer, high TIGIT expression on CD8^+^ TILs at the gene and protein level corresponded to high cell‐surface TIGIT expression on peripheral CD8^+^ T cells.^9^ Single‐cell RNA sequencing with paired T‐cell receptor (TCR) sequencing in 10 patients with gastric cancer identified a CD8^+^ T‐cell cluster with an effector memory (EM) phenotype with shared TCRs in the tumour and blood; this subset was enriched for migration‐related gene signatures, including CX3CR1 and several granzyme‐encoding genes, suggesting migratory abilities from the blood into tumour.^10^ An extensive single‐cell T‐cell atlas from multiple cancer types composed of tumour, adjacent normal tissue, and peripheral blood reported that potentially tumour‐reactive T cells were of an EM phenotype that expressed CD45RA (EMRA).^11^ In this study, modeling suggested a high degree of mobility between the circulation and tumour of EMRA T cells. In functional assays in which T cells expanded from PD‐1^+^ and PD‐1^‒^ peripheral blood lymphocytes were incubated with autologous tumour cells from patients with metastatic melanoma, hepatocellular carcinoma (HCC), renal cell carcinoma (RCC), or non‐small‐cell lung cancer (NSCLC), IFN‐γ release was markedly increased in the PD‐1^+^ lymphocyte fraction, indicating enrichment of tumour‐reactive T cells in PD‐1^+^ cells.^12^

Shared TCRs between intratumoural regulatory T cells (Tregs) and those in the periphery have also been documented. In patients with breast cancer, there was considerable overlap in intratumoural Treg TCR clonotypes with peripheral CD45RA^−^FOXP3^hi^ Tregs, suggesting movement of Tregs from one compartment to the other.^13^ In this study, the authors found that these peripheral Tregs were phenotypically similar to intratumoural Tregs, particularly in terms of expression of the chemokine receptor CCR8. Tregs were overrepresented as a fraction of CD4^+^ T cells in the periphery, tumour, and lymph nodes in patients with breast and pancreatic cancers compared to a control, suggesting that elevations in peripheral Tregs may reflect increases in intratumoural Tregs.^14^ A small trial of chemotherapy combined with immunotherapy (chemoIO) in patients with previously untreated RCC demonstrated shared clones between enriched peripheral CD8^+^ T cells and TILs.^15^ This study reported possible trafficking of tumour‐specific CD8^+^ T cells from the periphery into the tumour lesion using HLA‐A2 dextramers loaded with RCC tumour antigen. Overlapping T‐cell clonotypes between tumour and peripheral blood were identified in a patient with NSCLC. where clones found within TILs increased in frequency in the periphery after chemoIO.^16^ In a phase I study of the immunocytokine NHS‐IL12 in patients with metastatic solid tumours, TIL density and diversity were increased in tumour lesions of patients with a more robust IFN‐γ response; importantly, T‐cell clones expanded within the tumour were also detected in paired peripheral blood, and vice versa.^17^

In a study of 20 resectable oesophageal adenocarcinoma patients treated with neoadjuvant chemoradiotherapy plus anti‐PD‐1 (αPD‐1) therapy prior to surgery, higher frequencies of peripheral blood Tregs, CD14^+^CD16^+^ intermediate monocytes, type‐2 conventional dendritic cells, and early myeloid‐derived suppressor cells (MDSCs) at baseline in patients without a pathological complete response (pCR) were in concordance with enrichment in hallmark gene sets related to immunosuppression in paired baseline biopsies.^18^ Here, an association between tumour epithelial‐to‐mesenchymal (EMT) gene signature and frequencies of circulating Tregs and proliferating CD8^+^ T cells (expressing Ki67) was observed, suggesting that EMT‐associated immunosuppression in the tumour may be reflected in blood. In a cohort of 58 patients with advanced gastric cancers, patients with higher blood neutrophil‐to‐lymphocyte ratio (NLR) had enrichment of neutrophil‐related gene signatures and NLR in the tumour.^19^ Peripheral monocyte‐to‐lymphocyte ratios (MLR) reflected ratios of intratumoural M2‐like macrophages (expressing CD163) to CD8^+^ TILs in urothelial carcinoma (UC).^20^

Inferring tumour immune status based on evaluations of the peripheral immunome through liquid biopsies is undeniably complex. However, the studies discussed thus far highlight the potential overlap in immunological features between the TME and the periphery. They serve as a rationale for exploring the systemic immune system for biomarkers that are associated with clinical response to immunotherapies.

## 3 CIRCULATING CYTOKINES AND SOLUBLE PROTEINS

The interferon family of cytokines is pivotal in orchestrating pro‐inflammatory and anti‐tumour immune responses, and influences of the type 2 IFN‐γ in the TME have been reviewed at length.^21^ Despite this well‐known role, its potential utility as a biomarker in immunotherapy settings remains unclear. Induction of IFN‐γ and increased cellular responsiveness to IFN‐γ signalling have been correlated with improved outcomes, suggesting that downstream effects of signalling may be an avenue to explore in biomarker discovery.^22^, ^23^, ^24^ Table 1 summarizes circulating serum and plasma analytes discussed in this section.

**TABLE 1:** Circulating cytokine and soluble proteins associated with response.

Immune correlate	Cancer, stage (*n*)	Treatment	Direction/timepoint	Association with clinical outcome			Reference
				Response	PFS	OS	
IL‐6	NSCLC, R/M (*n* = 45)	αPD‐1	**↑** Baseline		**↓** (*p* < 0.05)		25
	NCSLC, R/M (*n* = 100)	αPD‐1	**↑** Baseline		**↓** (*p* < 0.01)		26
	NSCLC, advanced (*n* = 125)	αPD‐1, αPD‐L1	**↑** Baseline	**↓** (*p* < 0.01)	**↓** (*p* < 0.001)	**↓** (*p* < 0.001)	27
	SCLC (*n* = 37)	αCTLA‐4	**↑** Baseline			**↓** (*p* < 0.05)	28
	HCC, unresectable (*n* = 165)	αPD‐L1	**↑** Baseline	**↓** (*p* < 0.05)	**↓** (*p* < 0.01)	**↓** (*p* < 0.05)	29
	RCC, advanced (*n* = 58)	αPD‐1	**↑** Baseline	**↓** (*p* < 0.01)			30
	HCC, advanced (*n* = 64)	αPD‐L1	**↑** Baseline	**↓** (*p* < 0.05)	**↓** (*p* < 0.05)	**↓** (*p* < 0.05)	31
IL‐6^a^	NSCLC (*n* = 47)	αPD‐1	**↓** on tx		**↑** (*p* < 0.05)		32
IL‐10	RCC, advanced (*n* = 69)	αPD‐1, αCTLA‐4	**↑** Baseline		**↓** (*p* < 0.01)	**↓** (*p* < 0.001)	34
	Lung (*n* = 229)	αPD‐1, αCTLA‐4	**↑** Baseline		**↓** (*p* < 0.001)		35
IL‐8	UC, metastatic (*n* = 88)	αPD‐L1	**↑** Baseline	**↓** (*p* < 0.05)		**↓** (*p* < 0.05)	37
	UC, metastatic (*n* = 241)	αPD‐L1	**↑** Baseline	**↓** (*p* < 0.05)		**↓** (*p* < 0.01)	
	UC, metastatic (*n* = 451)	αPD‐L1	**↑** Baseline			**↓** (*p* < 0.001)	
	RCC, metastatic (*n* = 84)	αPD‐L1	**↑** Baseline			**↓** (*p* < 0.05)	
	RCC (*n* = 392)	αPD‐1	**↑** Baseline			**↓** (*p* < 0.0001)	38
	Squamous NSCLC (*n* = 108)	αPD‐1	**↑** Baseline			**↓** (*p* < 0.01)	
	non‐squamous NSCLC (*n* = 255)	αPD‐1	**↑** Baseline			**↓** (*p* < 0.0001)	
IL‐8^a^	UC, metastatic (*n* = 88)	αPD‐L1	**↑** On tx	**↓** (*p* < 0.05)		**↓** (*p* < 0.01)	37
	UC, metastatic (*n* = 241)	αPD‐L1	**↑** On tx	**↓** (*p* < 0.05)		**↓** (*p* < 0.05)	
	UC, metastatic (*n* = 297)	αPD‐L1	**↑** On tx	**↓** (*p* < 0.001)		**↓** (*p* < 0.01)	
sPD‐L1	NSCLC (*n* = 233)	αPD‐1	**↑** Baseline	**↓** (*p* < 0.05)	**↓** (*p* < 0.05)	**↓** (*p* < 0.001)	41
	NSCLC (*n* = 39)	αPD‐1	**↑** Baseline	**↓** (*p* < 0.01)	**↓** (*p* < 0.05)	**↓** (*p* < 0.05)	42
	gastric cancer (*n* = 439)	αPD‐1	**↑** Baseline		**↓** (*p* < 0.01)	**↓** (*p* < 0.001)	43
	NSCLC (*n* = 51)	αPD‐1	**↑** Baseline		**↓** (*p* < 0.01)		44
	NSCLC (*n* = 122)	αPD‐1, αPD‐L1	**↑** Baseline		**↓** (*p* < 0.01)	**↓** (*p* < 0.01)	45
	metastatic RCC (*n* = 43)	αPD‐1	**↑** Baseline	**↓** (*p* < 0.05)		**↓** (*p* < 0.05)	46
sPD‐1	gastric cancer (*n* = 439)	αPD‐1	**↑** Baseline			↓ (*p* < 0.05)	43
	NSCLC (*n* = 51)	αPD‐1	**↑** Baseline		**↓** (*p* < 0.01)		44
Granzyme B	NSCLC, stage IV (*n* = 78)	αPD‐1	**↑** Baseline		**↑** (*p* < 0.05)	**↑** (*p* < 0.05)	48

^a^ Studies examining the change in indicated immune correlate.

Abbreviations: chemoIO, chemoimmunotherapy; HCC, hepatocellular carcinoma; NSCLC, non‐small‐cell lung cancer; On tx, on treatment; OS, overall survival; PFS, progression‐free survival; R/M, recurrent and/or metastatic; RCC, renal cell carcinoma; RT, radiotherapy; SCLC, small cell lung carcinoma; UC, urothelial carcinoma.

### 3.1 Cytokines

Several biomarker identification studies have found potential prognostic utility of circulating IL‐6. Across two studies of patients with recurrent or advanced NSCLC receiving αPD‐1 agents, patients with higher plasma IL‐6 concentrations (>6.37 pg/mL or >11.15 pg/mL, respectively) had shorter progression‐free survival (PFS).^25^, ^26^ In 125 patients also with advanced NSCLC treated with αPD‐(L)1 inhibitors, baseline serum concentrations of IL‐6 ≥13.1 pg/mL were associated with shorter median overall survival (mOS; 7.4 months vs. not reached at 20 months).^27^ In this study, baseline IL‐6 was a better predictor of clinical response to αPD‐(L)1 inhibitors than tumour PD‐L1 expression, and also associated with OS in subgroup analyses of patients with no/low tumour PD‐L1 expression. Similar results were seen in patients with SCLC treated with chemotherapy with αCTLA‐4, where baseline serum IL‐6 levels >3.85 pg/mL were associated with shorter mOS (9.5 vs. 18.5 months).^28^ In 165 patients with unresectable HCC receiving chemoIO with αPD‐L1, baseline serum IL‐6 concentrations were significantly lower in patients achieving complete response (CR), partial response (PR), or stable disease (SD) >6 months (5.1 pg/mL) compared with those with progressive disease (PD) (11.6 pg/mL).^29^ Here, serum IL‐6 concentrations >18.49 pg/mL portended shorter median PFS (mPFS; estimated 1.5 vs. 7.0 months) and mOS (estimated 8.0 vs. not reached at 12 months) in the discovery cohort consisting of 84 patients. Similar results were seen in the validation cohort of 81 additional patients (mPFS: estimated 2.2 vs. 4.1 months; mOS: estimated 6.1 vs. not reached at 12 months) based on baseline IL‐6 stratification.^29^ In patients with advanced RCC receiving chemoIO, 72% with baseline serum IL‐6 < 6.5 pg/mL did not have disease progression at 12 months, whereas 41% with high serum IL‐6 remained progression‐free at the same timepoint.^30^ Plasma IL‐6 levels were identified as an independent prognostic factor in another cohort of advanced HCC patients (*n* = 64) treated with chemoIO and αPD‐L1, with high IL‐6 portending shorter PFS and OS.^31^

While baseline plasma concentrations were not associated with survival in another cohort of NSCLC patients (*n* = 47) treated with αPD‐1 agents, the change in plasma IL‐6 at an early timepoint during therapy compared with baseline was associated with PFS.^32^ Here, patients with a ≥40% decrease in IL‐6 had longer mPFS (10.6 months) compared to patients with stable IL‐6 (between 40% decrease and 40% increase, 5.1 months) or a 40% increase (4.2 months). Given the consistency of the association between IL‐6 and outcome with immunotherapy, continued evaluation of IL‐6 as a predictive and/or prognostic biomarker in solid tumours treated with ICI is warranted.

A few studies have highlighted the potential of IL‐10, a cytokine important in regulating inflammatory responses, as a circulating biomarker.^33^ In 69 patients with advanced RCC receiving first‐line ICI, those with baseline serum IL‐10 >4.3 ng/mL (*n* = 9) had significantly shorter mPFS (5.2 months vs. not reached at 30 months) and mOS (13.9 months vs. not reached at 30 months) than patients with levels <4.3 ng/mL (*n* = 60) in multi‐variate analyses, underscoring IL‐10 as an independent prognostic factor of survival.^34^ A recent meta‐analysis of 24 studies evaluated associations between peripheral cytokines with PFS and OS; in pooled univariate analyses including 229 patients with lung cancer treated with either αPD‐1 or αCTLA‐4 inhibitors, high serum IL‐10 was significantly associated with shorter PFS.^35^

### 3.2 IL‐8

**FIGURE 2:**
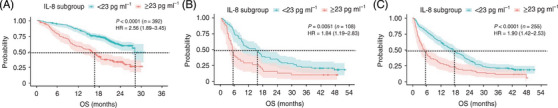
Association between baseline serum IL‐8 level and survival in patients treated with anti‐PD‐1 inhibitors. (A–C) Exploratory Kaplan–Meier analysis of the OS probability with 95% confidence intervals in patients with (A) RCC, (B) squamous NSCLC, and (C) nonsquamous NSCLC treated with anti‐PD‐1 blockers in prospective phase 3 clinical trials based on a baseline stratification of serum IL‐8 (23 pg/mL cutoff). The dotted horizontal lines indicate the 50% probability of OS, and the dotted vertical lines indicate median OS in high and low IL‐8 groups. NSCLC, non‐small‐cell lung cancer; OS, overall survival; RCC, renal cell carcinoma. *Source*: Adapted from Schalper et al.^38^ Copyright © 2020, under exclusive license to Springer Nature America, Inc.

IL‐8 (CXCL8) is arguably the most described chemokine in the context of cancer immunotherapy and is implicated in the promotion of tumour progression through the recruitment of immunosuppressive and pro‐tumourigenic cell populations.^36^ A large study of plasma IL‐8 as a prognostic biomarker in three clinical trials of αPD‐L1 in UC and RCC (*n* = 1445 patients total) consistently demonstrated that high baseline IL‐8 levels (above the median of the cohort, 15 pg/mL) were associated with shorter OS.^37^ For example, in 88 patients with locally advanced or metastatic UC treated with first‐line αPD‐L1, baseline plasma IL‐8 >15 pg/mL led to mOS of 6.3 months versus not reached at 25 months in the IL‐8 low group. With the same treatment except in the 2^+^ line in another cohort of 421 patients with UC, the same effects were seen between high and low‐baseline IL‐8 groups (mOS: 3.9 vs. 15.5 months). In this latter cohort, increases in plasma IL‐8 of ≥1.09 pg/mL at a set timepoint during treatment compared to baseline also were associated with shorter mOS (7.9 vs. 16.5 months).^37^ These findings were confirmed within the same study with 84 patients with metastatic RCC given αPD‐L1, where again high baseline plasma IL‐8 signified shorter mOS (30.2 months vs. not reached at 40 months). In another retrospective analysis of 755 patients with advanced cancers (RCC (*n* = 392), squamous NSCLC (*n* = 108), or nonsquamous NSCLC (*n* = 255)) receiving αPD‐1 agents, baseline levels of serum IL‐8 > 23 pg/mL were uniformly associated with significantly shorter OS (Figure 2).^38^ A pooled meta‐analysis of 24 studies through early 2022 (including some studies discussed above), comprising heterogeneous groups of solid tumours receiving αPD‐1 (with αCTLA‐4 in one study), confirmed the independent prognostic ability of peripheral IL‐8, with higher levels uniformly predicting shorter PFS and OS.^35^

### 3.3 sPD‐1 and sPD‐L1

Many signal transduction proteins, including canonical immune checkpoints, exist both in membrane‐bound and soluble forms, which may portend different biological outcomes.^39^, ^40^ Most of the existing literature has focused on soluble PD‐L1 (sPD‐L1) and suggests a relationship between elevated baseline sPD‐L1 and poor response to ICI. Among 233 NSCLC patients treated with αPD‐1 agents, those with elevated baseline serum sPD‐L1 (≥90 pg/mL) had shorter mPFS (57 vs. 177 days) and mOS (182 days vs. not reached at 1200 days).^41^ In a smaller study of NSCLC patients (*n* = 39) treated with αPD‐1, higher baseline plasma sPD‐L1 (>3.36 ng/mL) correlated with shorter mOS (7.20 months vs. not reached at 14 months) and shorter time to treatment failure (1.48 vs. 5.36 months).^42^ Here, patients with high baseline sPD‐L1 also exhibited a worse overall response rate (ORR), as only 25% (vs. 59% among patients with low baseline sPD‐L1) achieved a CR or PR, and 75% (vs. 22%) developed PD.^42^ This association has also been observed in other cancer types. In 439 patients with gastric cancer, those with elevated baseline plasma sPD‐L1 (>295 or >286 pg/mL) had shorter mOS (4.1 vs. 8.9 months) and mPFS (1.7 vs. 2.1 months) upon treatment with αPD‐1 agents.^43^ High baseline sPD‐L1 also predicted poor response to ICI in other studies of patients with NSCLC and metastatic RCC.^44^, ^45^, ^46^ There is also general consensus on the correlation between elevated baseline sPD‐1 and poor response to ICI in patients with NSCLC or gastric cancer.^43^, ^44^

### 3.4 Granzyme B

Granzyme B is a key serine protease and cytolytic effector secreted by activated T cells and NK cells that initiates apoptosis in target cells.^47^ In 78 patients with stage IV NSCLC receiving αPD‐1 therapy, baseline serum granzyme B <1.27 pg/mL was associated with worse mPFS (estimated 75 vs. 225 days) and mOS (estimated 225 vs. 500 days) compared to those with levels above this threshold. Patients with low granzyme B also had reduced frequencies of peripheral CD8^+^ EMRA (44% vs. 54%) and PD‐1^+^TIM3^+^CD8^+^ (4.5% vs. 7.8%) T cells.^48^ Importantly, prior chemotherapy may impact granzyme B concentrations, as seen in a cohort of patients with gastric cancer, where those receiving prior chemotherapy (*n* = 49) had significantly lower mean serum granzyme B levels compared to chemotherapy‐naive patients (*n* = 38) (9.68 vs. 37.93 pg/mL, respectively).^49^ Whether changes in peripheral granzyme B concentrations in response to immunotherapy impact outcome remains unknown. However, it has been shown that treatment with the immunocytokine NHS‐IL12 increases serum granzyme B levels in patients with advanced cancers 2 weeks after treatment, which is indicative of T‐cell and/or NK‐cell cytolytic behavior.^50^

## 4 T CELLS

Monitoring peripheral T cells in terms of frequency, phenotype, and clonality over the course of immunotherapy is an attractive arena for biomarker discovery. However, this may be a double‐edged sword, as their sheer diversity may preclude a reliable biomarker. Candidate peripheral T‐cell biomarkers are summarized in Table 2. Cancer neoantigen‐specific T cells have been identified within the PD‐1^+^ fraction in many studies^12^, ^51^; however, PD‐1 is a broad marker of activation and by itself does not indicate recognition of tumour. Increased frequencies of Ki67 expression on T cells after ICI are suggestive of a proliferative burst, and Ki67^+^ expression by CD8^+^ T cells has been shown to be associated with prolonged survival in multiple cohorts of patients treated with αPD‐1 therapy. Patients with recurrent/metastatic (R/M) thymic epithelial tumours (*n* = 31) or metastatic NSCLC (*n* = 33 in discovery set and *n* = 46 in validation) with a ≥2.8‐fold increase of CD8^+^ Ki67^+^ T cells 1 week after first treatment compared to baseline had improved response and disease control, longer PFS, and/or longer OS.^52^ Associations between the circulating TCR repertoire and response to immunotherapy have also been evaluated in several studies; however, it is evident that clonal composition at baseline prior to immunotherapy may influence outcome differently based on the ICI agent used.^53^, ^54^, ^55^ T‐cell clonality within more phenotypically defined populations may hold promise as a candidate biomarker. In stage IIIB‐IV, NSCLC patients (*n* = 40) treated with αPD‐(L)1, higher diversity within the circulating PD‐1^+^ CD8^+^ T‐cell fraction prior to the start of therapy was associated with longer mPFS (6.4 vs. 2.5 months) and mOS (not reached at 25 vs. 10.1 months).^56^

**TABLE 2:** Peripheral T‐cell subsets associated with response.

Immune Correlate	Cancer, stage (*n*)	Treatment	Direction/timepoint	Association with clinical outcome			Reference
				Response	PFS	OS	
CD8^+^ PD‐1^+^ Ki67^+^ T cells^a^	Thymic epithelial tumours, R/M (*n* = 31)	αPD‐1	**↑** On tx		**↑** (*p* < 0.05)		52
	NSCLC, metastatic (*n* = 33)	αPD‐1	**↑** On tx		**↑** (*p* < 0.01)	**↑** (*p* < 0.01)	
	NSCLC, metastatic (*n* = 46)	αPD‐1	**↑** On tx	**↑** (*p* < 0.05)	**↑** (*p* < 0.01)	**↑** (*p* < 0.05)	
CD8^+^ PD‐1^+^ T cell diversity	NSCLC, stage IIIB‐IV (*n* = 40)	αPD‐(L)1	**↑** Baseline		**↑** (*p* < 0.01)	**↑** (*p* < 0.05)	56
CD8^+^ PD‐1^+^ TIGIT^+^ T cells	NSCLC, R/M (*n* = 263)	αPD‐(L)1	**↑** Baseline		**↓** (*p* < 0.001)	**↓** (*p* < 0.05)	57
CD8^+^ PD‐1^+^ TIGIT^+^ T cells^a^	Merkel cell carcinoma, R/M (*n* = 15)	αPD‐1	**↑** On tx	**↑** (*p* < 0.05)		**↑** (*p* < 0.01)	58
LAG3^+^ T cells	UC (*n* = 94)	αPD‐1 +/− αCTLA‐4	**↑** Baseline	**↓** (*p* < 0.01)	**↓** (*p* < 0.01)	**↓** (p < 0.001)	59
4‐1BB^+^ T cells	HNSCC, R/M (*n* = 40)	αPD‐1 +/− chemoIO	**↑** Baseline		**↑** (*p* < 0.05)	**↑** (*p* < 0.05)	60
CD8^+^ CD57^+^ CD28^−^ KLRG1^+^ T cells	NSCLC, advanced (*n* = 83)	αPD‐(L)1	**↑** Baseline	**↓** (*p* < 0.05)	**↓** (*p* < 0.01)	**↓** (*p* < 0.05)	61
CD8^+^ CXCR31^+^ T cells^a^	NSCLC (*n* = 29)	αPD‐(L)1	**↑** On tx	**↑** (*p* < 0.05)	**↑** (*p* < 0.01)	**↑** (*p* < 0.05)	16
	NSCLC (*n* = 36)	αPD‐1	**↑** On tx	**↑** (*p* < 0.05)	**↑** (*p* < 0.01)	**↑** (*p* < 0.05)	62

^a^ Studies examining the change in indicated immune correlate.

Abbreviations: chemoIO, chemoimmunotherapy; HNSCC, head and neck squamous cell carcinoma; mAb, monoclonal antibody; NSCLC, non‐small cell lung cancer; On tx, on treatment; OS, overall survival; PFS, progression‐free survival; R/M, recurrent/metastatic; UC, urothelial carcinoma.

### 4.1 T cells expressing immune checkpoints in addition to PD‐1

**FIGURE 3:**
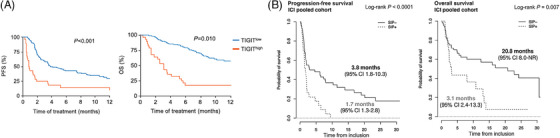
Associations between severely exhausted or senescent circulating CD8^+^ T cells with survival during anti‐PD‐(L)1 therapy. (A) Kaplan–Meier analyses of PFS (left) and OS (right) in patients with recurrent and/or metastatic NSCLC based on low or high TIGIT expression by peripheral CD8^+^ PD‐1^+^ T cells. *Source*: Figure adapted from Kim et al.^57^ Copyright © 2019. Published by Elsevier Ltd. All rights reserved. (B) Kaplan–Meier analyses of PFS (left) and OS (right) probability in patients from pooled training and validation cohorts with advanced NSCLC receiving single‐agent anti‐PD‐(L)1 inhibitor. Patients were stratified by senescent immune phenotype (SIP; CD57^+^ KLRG1^+^ CD28^−^ as a percentage of peripheral CD8^+^ T cells), where cutoff was determined by maximization of the log‐likelihood ratio method. NSCLC, non‐small cell lung cancer; OS, overall survival; PFS, progression‐free survival. *Source*: Adapted from Ferrara et al.^61^ Copyright © 2021, American Association for Cancer Research.

T‐cell expression of other immune checkpoints in addition to PD‐1 may be useful in evaluating response to immunotherapy, with co‐expression of PD‐1 and TIGIT on T cells particularly warranting further evaluation. Higher baseline frequencies of CD8^+^ PD‐1^+^ TIGIT^+^ T cells were associated with shorter mPFS (estimated 1.0 vs. 4.0 months) and mOS (estimated 4.0 vs. not reached at 12 months) in advanced NSCLC patients (*n* = 263) receiving αPD‐(L)1 therapies (Figure 3A).^57^ Greater frequencies of peripheral T cells co‐expressing PD‐1 and TIGIT 3 weeks after the start of αPD‐1 therapy in Merkel cell carcinoma patients (*n* = 15) were also associated with improved BOR.^58^ The pattern of LAG‐3 expression on T cells also holds promise as a biomarker in patients with UC. Patients with a prominent LAG‐3‐negative expression pattern demonstrated longer mOS (27.6 months) than patients with a LAG‐3^+^ T‐cell phenotype (4.7 months).^59^ Longer PFS was also observed in the LAG‐3‐negative group in this study. Higher expression of 4‐1BB by peripheral T cells at baseline (<1.65% of T cells) led to improved response and longer PFS (not reached at 20 vs. 2.5 months) and OS (not reached at 25 vs. 3.5 months) in recurrent and metastatic head and neck squamous cell carcinoma (HNSCC) patients receiving αPD‐1 therapy with or without concurrent chemotherapy (*n* = 40).^60^

Immunosenescent T cells prior to ICI may have a role in predicating response. Higher frequencies of circulating CD8^+^ T cells with a CD57^+^ CD28^−^ KLRG1^+^ phenotype, indicating late‐stage differentiation and immunosenescence, were strongly associated with worse response, mPFS (1.7 vs. 3.8 months), and mOS (3.1 vs. 20.8 months) after multivariate regression in advanced NSCLC patients given single‐agent αPD‐(L)1 in both discovery and validation cohorts (Figure 3B).^61^ Chemokine receptor expression on peripheral T cells has also been examined for association with outcome in response to immunotherapy. While baseline levels of CX3CR1^+^ CD8^+^ T cells were not associated with outcome in two cohorts of NSCLC patients treated with chemoIO with αPD‐(L)1 agents (*n* = 65 cumulatively), the percentage change in the frequency of CX3CR1^+^ CD8^+^ T cells, which varied between 10% to 20% between studies, could distinguish patients with PR from those with SD/PD. This increase in CX3CR1^+^ CD8^+^ T cells was associated with improved ORR as early as 4 weeks after treatment initiation with chemoIO and highly correlated with ORR at 9 weeks.^16^, ^62^

### 4.2 Tregs

Peripheral Tregs are rare circulating T cells, and existing data on frequencies and kinetics during ICI remain sparse. Peripheral Treg frequencies, when considered together with frequencies of additional conventional CD4^+^ T‐cell subsets, may offer predictive abilities. In a cohort of 126 patients with NSCLC, a prediction formula incorporating CD62L^lo^ CD4^+^ T cells and CD25^+^ Foxp3^+^ Tregs at baseline could discriminate between patients with a BOR of CR, PR, or SD and those with PD at 9 weeks after start of αPD‐1 therapy.^63^ Peripheral Tregs may also be useful in resolving whether very early tumour growth patterns are reflective of true progression or pseudoprogression. In a study of 74 patients with advanced NSCLC receiving αPD‐(L)1 inhibitors, 10 patients experienced pseudoprogression at 7 days post‐treatment initiation (defined as early progression but PR or SD at the first response assessment after 3−4 cycles of treatment). These patients had significantly decreased frequencies of circulating CD4^+^CD25^+^CD127^lo^Foxp3^+^ Tregs (as a percentage of total CD4^+^ T cells) at day 7 vs. baseline. Conversely, circulating Tregs increased in six patients who experienced hyperprogression on day 7 (confirmed PD at first response evaluation).^64^ As peripheral Tregs may very well be a major source of intratumoural Tregs, continued study of their peripheral kinetics in relationship to immunotherapy remains worthwhile.^13^

## 5 B AND NK CELLS

B cells within tertiary lymphoid structures in the TME have been associated with improved response to immunotherapy.^65^, ^66^ However, the few studies examining circulating B cells as biomarkers for cancer immunotherapy report conflicting associations (Table 3). In a heterogeneous cohort of 78 patients with solid tumours, patients with a BOR of PR (*n* = 36) or SD (*n* = 18) had significantly reduced frequencies of peripheral B cells immediately prior to the start of αPD‐1 immunotherapy compared with patients with PD (*n* = 36).^67^ In a cohort of 150 patients with advanced NSCLC, patients who experienced PR or SD within 180 days of αPD‐1 initiation (*n* = 17) had greater baseline frequencies of CD19^+^ B cells than patients developing PD (*n* = 33).^68^ In this cohort, higher baseline frequencies of IgM^+^ CD27^+^ memory B cells were associated with longer PFS. While baseline B cell frequencies were not associated with ORR in another heterogeneous cohort of 39 patients with solid tumours, increases in frequencies of a more specific B cell phenotype, CD27^‒^ IgD^+^ IgM^+^ naïve B cells, at first response evaluation were associated with a BOR of CR, PR, or SD, while increases in immature CD21^‒^ B cells correlated with worse outcome.^69^

**TABLE 3:** Peripheral B‐ and NK‐cell subsets associated with response.

Immune correlate	Cancer, stage (*n*)	Treatment	Direction/timepoint	Association with clinical outcome			Reference
				Response	PFS	OS	
CD19^+^ B cells	Solid tumours (*n* = 78)	αPD‐1	**↑** Baseline	**↓** (*p* < 0.001)			67
CD19^+^ B cells	NSCLC, advanced (*n* = 150)	αPD‐1	**↑** Baseline	**↑** (*p* < 0.01)			68
IgM^+^ CD27^+^ memory B cells			**↑** Baseline	**↑** (*p* < 0.01)	**↑** (*p* < 0.01)		
CD27^−^ IgD^+^ IgM^+^ naïve B cells^a^	Solid tumours (*n* = 39)	ICI, unspecified	**↑** On tx	**↑** (*p* < 0.05)			69
CD21^−^ B cells			**↑** On tx	**↓** (*p* < 0.05)			
CD3^−^ CD56^+^ NK cells^a^	NSCLC (*n* = 40)	αPD‐1	**↑** On tx	**↑** (*p* < 0.001)			70
CD56dim CD16^+^ NK cells	NSCLC, advanced (*n* = 65)	αPD‐1	**↑** Baseline		**↑** (*p* < 0.01)		71
CD3‐CD56^+^ NK cells^a^	HCC, advanced (*n* = 61)	αPD‐(L)1	**↑** On tx		**↑** (*p* < 0.05)	**↑** (*p* < 0.05)	72
CD56+ PD‐1^+^ NK cells	NSCLC (*n* = 55)	αPD‐(L)1	**↑** Baseline			**↑** (*p* < 0.05)	73
CD25^+^ NK cells^a^	NSCLC, R/M (*n* = 24)	αPD‐(L)1	**↑** On tx	**↑** (*p* < 0.01)			74

^a^ Studies examining the change in indicated immune correlate.

Abbreviations: HCC, hepatocellular cancer; ICI, immune checkpoint inhibitor; NK, natural killer; NSCLC, non‐small cell lung cancer; On tx, on treatment; OS, overall survival; PFS, progression‐free survival; R/M, recurrent/metastatic.

Studies that have examined frequencies of peripheral NK cells in patients with solid tumours are near unanimous in reporting that NK cells are associated with better outcomes with immunotherapy (Table 3). In NSCLC patients with disease recurrence or progression after chemotherapy, patients with a BOR of CR, PR, or SD for at least 6 months (*n* = 25) after treatment with an αPD‐1 inhibitor had significantly increased frequencies of peripheral CD3^‒^CD56^+^ NK cells after αPD‐1, an effect not seen in patients with PD or SD less than 6 months duration (*n* = 15).^70^ In another treatment‐naive cohort of 65 NSCLC patients with stage IIIB/IV disease who went on to receive first‐line αPD‐1 therapy, starting frequencies of CD3^‒^CD56^dim^CD16^+^ NK cells were significantly correlated with prolonged PFS.^71^ While baseline frequencies of CD3^‒^CD56^+^ NK cells were not associated with outcome in 61 patients with advanced and/or metastatic HCC treated with αPD‐(L)1 inhibitors, changes in NK‐cell frequencies after treatment were independent prognostic factors for survival, with higher levels of CD3^‒^CD56^+^ NK cells positively associating with prolonged PFS and OS.^72^ A few studies have also found correlations between an activated phenotype of NK cells and outcome. In cohorts of advanced NSCLC with no prior ICI exposure treated with αPD‐(L)1 inhibitors, higher frequencies of circulating CD56^+^ PD‐1^+^ NK cells at baseline were associated with improved OS and greater CD25 (IL2Rα) expression on CD56^+^ NK cells after two cycles of chemoIO was associated with clinical benefit.^73^, ^74^

## 6 MYELOID CELL POPULATIONS

Immune cells of myeloid origin are largely innate cells with pro‐tumour and anti‐tumour influences within the TME and periphery and have been explored as biomarkers.^75^ Absolute neutrophil, monocyte, eosinophil, and lymphocyte counts (ANC, AMC, AEC, and ALC, respectively) can be easily obtained from peripheral blood during routine patient care, enabling frequent assessment. Although peripheral myeloid cells are not universally used as prognostic indicators in the clinic, a growing body of research demonstrates their potential to predict cancer patient response to immunotherapy.

### 6.1 Neutrophils

While neutrophils can have both pro‐tumourigenic and anti‐tumourigenic functions, most studies show negative associations between peripheral neutrophils and patient outcomes. Neutrophils can support tumour growth through mechanisms including the release of reactive oxygen species, activation of tumour‐promoting signalling pathways, release of neutrophil extracellular traps, and T‐cell exclusion from the TME.^76^ Systemically, neutrophil numbers and function can be influenced by tumours through activation of granulopoiesis by tumour‐released factors and alteration of metabolic activity in neutrophils.^76^ Moreover, peripheral neutrophils can support metastatic spread of cancer cells through processes such as formation of neutrophil‐cancer cell clusters and development of premetastatic niches in distant organs.^76^

Numerous studies evaluate the prognostic abilities of ANC in the context of immunotherapy, with a few highlighted here (Table 4). In patients (*n* = 104) with various recurrent and/or metastatic cancers treated with αPD‐(L)1, higher ANC at baseline is associated with worse OS.^77^ In patients with locally advanced or metastatic pancreatic cancer (*n* = 122) treated with αPD‐(L)1 or αCTLA‐4, higher ANC (>3.3 × 10^9^/L) was predictive of worse OS and PFS.^78^ Higher baseline ANC was associated with worse OS and PFS in 186 patients with HNSCC treated with αPD‐1 as monotherapy or in combination with additional therapies.^79^ In 422 patients with metastatic RCC given αPD‐1 in the second line, higher baseline ANC was associated with worse OS, and a greater early post‐treatment increase of ANC (≥730 × 10^3^/L) was associated with shorter mOS (20.8 vs. 46.9 months) and mPFS (6.1 vs. 11.0 months).^80^ In a meta‐analysis of 38 studies of 4154 patients with multiple cancer types treated with αPD‐(L)1 or αCTLA‐4 therapies either alone or in combination with other treatments, patients with upward trends in NLR 4−8 weeks post‐treatment had worse OS, PFS, and tumour responses.^81^ Neutrophils are short‐lived cells and peripheral counts can therefore fluctuate. Factors which may influence these counts include immunosuppressive agents, chronic infections, or autoimmune disease. In addition, the timing of the baseline sampling prior to therapy may influence the count. These factors were not consistently discussed in each of the studies reviewed here; regardless, there is still a consistent negative association between neutrophil count and patient outcomes.

**TABLE 4:** Associations between peripheral neutrophils and monocytes with response.

Immune correlate	Cancer type, stage (*n*)	Treatment	Direction/timepoint	Association with clinical outcome			Reference
				Response	PFS	OS	
ANC	Various malignancies, advanced (*n* = 104)	αPD‐1, αPD‐L1	**↑** Baseline			**↓** (*p* < 0.01)	77
	Pancreatic, locally advanced, or metastatic (*n* = 122)	αPD‐1, αPD‐L1, αCTLA‐4	**↑** Baseline		**↓** (*p* < 0.001)	**↓** (*p* < 0.001)	78
	HNSCC (*n* = 186)	αPD‐1, αPD‐L1	**↑** Baseline		**↓** (*p* < 0.01)	**↓** (*p* < 0.01)	79
ANC^a^	RCC, advanced (*n* = 422)	αPD‐1	**↑** On tx	**↓** (*p* < 0.001)	**↓** (*p* < 0.05)	**↓** (*p* < 0.05)	80
NLR	Various malignancies, N/S (*n* = 4154)	αPD‐1, αPD‐L1, αCTLA‐4	**↑** Baseline	**↓** (*p* < 0.001)	**↓** (*p* < 0.001)	**↓** (*p* < 0.001)	81
NLR^a^	Various malignancies, N/S (*n* = 4154)	αPD‐1, αPD‐L1, αCTLA‐4	**↑** On tx		**↓** (*p* < 0.001)	**↓** (*p* < 0.001)	81
AMC	NSCLC, N/S (*n* = 157)	αPD‐1	**↑** Baseline		**↓** (*p* < 0.05)	**↓** (*p* < 0.05)	83
	ESCC, advanced (*n* = 43)	αPD‐1	**↑** Baseline			**↓** (*p* < 0.05)	84
	HPV+ malignancies, advanced (*n* = 65)	Bintrafusp alfa	**↑** Baseline	**↓** (*p* < 0.01)			85
MLR	Various solid malignancies, IIIB‐IV (*n* = 61)	αPD‐1	**↑** Baseline			**↓** (*p* < 0.05)	86
	CRC, advanced (*n* = 110)	αPD‐1, αPD‐L1	**↑** Baseline			**↓** (*p* < 0.001)	87
	ESCC, advanced (*n* = 162)	αPD‐1 + chemoIO	**↑** Baseline		**↓** (*p* < 0.05)	**↓** (*p* < 0.001)	88
PD‐L1+ monocytes	NSCLC (*n* = 35)	αPD‐1	**↑** Baseline	**↓** (*p* < 0.05)	**↓** (*p* < 0.05)	**↓** (*p* < 0.05)	89
CD14^low^ monocytes	Various solid malignancies, advanced (*n* = 89)	αPD‐1, αPD‐L1	**↑** Baseline			**↓** (*p* < 0.01)	77
Intermediate CD14^+^CD16^+^/HLA‐DR^++^ monocytes^a^	NSCLC, stage IIIB‐IV (*n* = 65)	αPD‐1	**↑** On tx		**↑** (*p* < 0.05)		71

^a^ Studies examining the change in indicated immune correlate.

Abbreviations: AMC, absolute monocyte count; ANC, absolute neutrophil count; CRC, colorectal cancer; ESCC, oesophageal squamous cell carcinoma; HNSCC, head and neck squamous cell carcinoma; HPV, human papillomavirus; MLR, monocyte‐to‐lymphocyte ratio; N/S, not specified; NLR, neutrophil‐to‐lymphocyte ratio; NSCLC, non‐small cell lung cancer; On tx, on treatment; OS, overall survival; PBMC, peripheral blood mononuclear cells; PFS, progression‐free survival; RCC, renal cell carcinoma; UC, urothelial cancer.

### 6.2 Monocytes

Monocytes can differentiate into tumour‐associated macrophages (TAMs) after migrating into the TME, which may contribute to an immunosuppressive microenvironment.^82^ Many studies have documented negative correlations between circulating monocytes and clinical outcomes with immunotherapy (Table 4). In advanced NSCLC patients (*n* = 157) and oesophageal squamous cell carcinoma patients (*n* = 43) treated with αPD‐1 inhibitors, high AMC (>630/μL and >650/μL) was significantly associated with worse OS.^83^, ^84^ Studies measuring circulating monocytes via flow cytometry in human papillomavirus (HPV)‐associated malignancies have shown concordant findings.^85^ Accordingly, baseline MLR has also been repeatedly linked to worse OS across multiple studies.^86^, ^87^, ^88^ PD‐L1 expression on peripheral monocytes may also influence response to immunotherapy. In 35 patients with lung cancer treated with αPD‐1 agents, high PD‐L1 expression on total monocytes as well as refined monocyte subpopulations predicted poor response to therapy. In this study, low PD‐L1 expression on monocytes correlated with improved mOS (17.06 vs. 6.83 months) and mPFS (16.00 vs. 4.62 months).^89^ CD14^lo^ monocytes also predicted worse OS in patients with various recurrent and/or metastatic malignancies treated with αPD‐(L)1 inhibitors.^77^

Studies have also evaluated the predictive and prognostic abilities of circulating monocytes after ICI. Levels of CD14^dim^CD16^+^ non‐classical monocytes, HLA‐DR^++^ nonclassical monocytes, and HLA‐DR^++^ intermediate monocytes at the time of first radiographic evaluation correlated with better PFS in a trial of αPD‐1 therapy as a first‐line treatment in patients with advanced NSCLC (*n* = 65) with <50% tumour expression of PD‐L1.^71^ While classical monocytes tend to extravasate into tumours where they can promote inflammation and form TAMs, nonclassical monocytes remove damaged cells and resolve inflammation in the vascular endothelium, which may explain their correlation with better survival. While most studies demonstrate a strong negative correlation between peripheral monocytes at baseline, changes in peripheral classical and nonclassical monocytes may be a dynamic predictor of clinical response following treatment with immunotherapy.

### 6.3 Eosinophils

Eosinophils have an anti‐tumourigenic role in many cancer types, in part due to the production of chemokines and cytokines which attract CD8+ T cells and skew macrophages to an M1 phenotype in the TME, although pro‐tumourigenic roles have also been identified.^90^ Eosinophils may also impact clinical outcomes in response to immunotherapy (Table 5). Baseline eosinophil counts of ≥220/µL were associated with shorter mOS (25 vs. 48 months) in a cohort of 37 patients with malignant pleural mesothelioma receiving immunotherapy; similar findings were noted in patients treated with chemoIO or chemotherapy alone.^91^ Alternatively, high levels of eosinophils at baseline or an increase with treatment have been positively associated with improved outcomes.^71^, ^92^, ^93^ In NSCLC patients treated with αPD‐(L)1 therapies, those with baseline eosinophil counts ≥130/µL had better response and longer mPFS (7.0 vs. 2.5 months) and mOS (9.0 vs. 5.5 months).^92^ The neutrophil‐to‐eosinophil ratio (NER), representative of systemic inflammation, was a negative prognostic factor at baseline in a cohort of metastatic RCC patients (*n* = 162) treated with αPD‐1or αCTLA‐4 agents.^94^, ^95^ Patients with metastatic RCC (*n* = 166) with a lower NER at baseline had a better BOR following ICI, with fewer patients developing PD and more developing a CR, PR, and SD than patients with a higher NER.^95^ In this study, a decrease in NER >50% at week 6 compared with baseline was associated with improved PFS and OS. These studies support the continued study of blood eosinophils as prognostic indicators of response and survival following immunotherapy.

**TABLE 5:** Associations between circulating eosinophils, DCs, and MDSCs with response.

Immune correlate	Cancer type, stage (*n*)	Treatment	Direction/timepoint	Association with clinical outcome			Reference
				Response	PFS	OS	
AEC	Malignant pleural mesothelioma (*n* = 37)	αPD‐1, αCTLA‐4	**↑** Baseline	**↓** (*p* < 0.05)	**↓** (*p* < 0.05)	**↓** (*p* < 0.01)	91
	NSCLC, advanced (*n* = 158)	αPD‐(L)1	**↑** Baseline	**↑** (*p* < 0.01)	**↑** (*p* < 0.01)	**↑** (*p* < 0.01)	92
NER	RCC, advanced (*n* = 162)	αPD‐1, αCTLA‐4	**↑** Baseline	**↓** (*p* < 0.05)	**↓** (*p* < 0.05)	**↓** (*p* < 0.01)	94
	RCC, advanced (*n* = 166)	αPD‐1, αCTLA‐4	**↑** Baseline	**↓** (*p* < 0.05)			95
NER^a^	RCC, advanced (*n* = 166)	αPD‐1, αCTLA‐4	**↑** On tx		**↓** (*p* < 0.05)	**↓** (*p* < 0.05)	95
total DC	Various solid malignancies, advanced (*n* = 89)	αPD‐1, αPD‐L1	**↑** Baseline			**↑** (*p* < 0.01)	77
pDC			**↑** Baseline			**↑** (*p* < 0.01)	
total DC	NSCLC (*n* = 35)	αPD‐1	**↑** Baseline	**↑** (*p* < 0.05)	**↑** (*p* < 0.05)	**↑** (*p* < 0.05)	97
MDSC	HPV+ malignancies, advanced (*n* = 65)	Bintrafusp alfa	**↑** Baseline	**↓** (*p* < 0.05)			85
PD‐L1+ MDSC			**↑** Baseline	**↓** (*p* < 0.05)			
M‐MDSC cells	NSCLC, IIIB‐IV (*n* = 132)	αPD‐1	**↑** Baseline		**↓** (*p* < 0.05)	**↓** (*p* < 0.01)	99
PMN‐MDSC cells			**↑** Baseline		**↓** (*p* < 0.05)	**↓** (*p* < 0.05)	

^a^ Studies examining the change in indicated immune correlate.

Abbreviations: AEC, absolute eosinophil count; HPV, human papillomavirus; mDC, myeloid dendritic cells; M‐MDSC, monocytic myeloid‐derived suppressor cells; NER, neutrophil‐to‐eosinophil ratio; NSCLC, non‐small cell lung cancer; On tx, on treatment; OS, overall survival; pDC, plasmacytoid dendritic cells; PFS, progression‐ free survival; PMN‐MDSC, polymorphonuclear MDSC; RCC, renal cell carcinoma.

### 6.4 Dendritic cells

Dendritic cells (DCs) encompass a diverse family of antigen‐presenting cells with crucial roles in T‐cell activation and memory response that can overcome an immunosuppressive TME.^96^ There is a relative consensus among studies on the positive correlation between peripheral blood DCs and patient outcomes (Table 5). In patients with recurrent and/or metastatic solid malignancies treated with αPD‐(L)1 inhibitors, higher total DC counts showed a trend toward improved OS, and many refined DC subsets including plasmacytoid DCs (pDCs) also showed significant positive associations.^77^ In patients with NSCLC (*n* = 35) treated with αPD‐1, patients with PFS ≤ 1 month had lower baseline percentages of total DC, pDC, CD1c^+^ myeloid DC (mDC), and CD141^+^ mDC compared to patients with PFS > 1 month or clinical response of SD, PR, or CR. Additionally, patients with lower total DCs had worse PFS and OS.^97^ In another study of patients with lung cancer (*n* = 35), higher absolute count of CD303^+^ pDCs, and frequencies of CD303^+^ pDCs, CD1c^+^ mDCs, and CD141^+^ mDCs as a percentage of leukocytes prior to αPD‐1 therapy corresponded with improved clinical response. However, higher PD‐L1 expression on DC subsets was associated with tumours.^89^ Overall, DCs tend to associate positively with clinical response, except DCs that express PD‐L1. Additional investigation, both at baseline and post‐treatment, will help to elucidate how pre‐therapy levels and changes in DCs following immunotherapy associate with patient outcomes.

### 6.5 Myeloid‐derived suppressor cells

MDSCs are immature myeloid cells that fail to differentiate into mature cells and instead acquire immunosuppressive functions.^98^ Studies regarding peripheral MDSCs and patient outcomes in response to immunotherapy all suggest a negative prognostic relationship (Table 5). In 65 patients with HPV‐associated malignancies treated with a dual inhibitor of PD‐L1 and TGFβ, patients developing PD had significantly higher baseline frequencies of total MDSCs and MDSCs expressing PD‐L1 than patients classified as responders.^85^ Furthermore, in patients with stage IIIB‐IV NSCLC (*n* = 132) receiving αPD‐1 as first‐line therapy, lower frequencies of monocytic MDSCs (M‐MDSCs) and polymorphonuclear MDSCs (PMN‐MDSCs) associated with improved mPFS (M‐MDSC, 5.2 vs. 2.3 months; PMN‐MDSC, 6.5 vs. 3.4 months) and mOS (M‐MDSC, 5.8 vs. 4.3 months; PMN‐MDSC, 7.0 vs. 4.3 months).^99^ The studies suggest that higher frequencies of total MDSCs and subsets of MDSCs at baseline are associated with worse responses to treatment. Whether this is a reflection of an immunosuppressive tumour microenvironment or some other measure is to be determined. Studies investigating changes in MDSCs after immunotherapy have not reported any significant findings with patient response; however, further investigation is warranted due to the small number of studies.^18^, ^85^, ^99^, ^100^

## 7 MODELING OF MULTIPLE IMMUNE PARAMETERS

The studies discussed thus far have for the most part examined individual peripheral immune correlates in a univariate manner. Few studies have examined the predictive and prognostic ability of ratios of peripheral cell subsets, other than NLR, MLR, and NER, for immunotherapy of solid tumours (Table 6). Two studies in breast cancer report on a peripheral immunoscore based on negative or positive contributions of multiple immune cell subsets, representing overall immune activation prior to therapy, that associates with patient outcomes in patients receiving immunotherapy plus chemotherapy. The peripheral immunoscore was calculated by dividing patients into tertile groups for each peripheral blood mononuclear cell (PBMC) subset (as % PBMC) and assigning points (0, 1, or 2) to the groups. A higher number was assigned to the high group subset if a positive effect on the anti‐tumour immune response was expected, and a higher number was assigned to the low subset group if an adverse effect on the anti‐tumour immune response was expected. The addition of points assigned to each PBMC subset for a given patient determined the peripheral immunoscore. In metastatic breast cancer patients treated with docetaxel ± PANVAC vaccine, a higher peripheral immunoscore at baseline representing enhanced immune activation associated with better mPFS (336 vs. 108 days) in patients receiving the combination therapy but not in patients receiving chemotherapy alone.^101^ Similarly, in patients with triple‐negative breast cancer receiving capecitabine, αPD‐1, or the combination, a similar peripheral immunoscore at baseline was associated positively with improved disease‐free survival for patients treated with immunotherapy but not chemotherapy alone.^102^

**TABLE 6:** Models incorporating multiple peripheral immune parameters.

Model	Contributors	Cancer, stage (*n*)	Treatment	Direction/timepoint	Association with clinical outcome			Reference
					Response	PFS	OS	
Peripheral immunoscore	CD4^+^ CM, CD8^+^ CM, T cells lacking immunosuppressive markers, CD49d‐ Tregs, CD56brightCD16‐ NK cells, early MDSCs	breast cancer, metastatic (*n* = 23)	docetaxel + PANVAC vaccine	↑ Baseline		**↑** (*p* < 0.001)		101
	PD‐1^+^ CD4, PD‐1^+^ CD8, CD4^+^ EM, CD8^+^ EM, ICOS^+^ Tregs, NKp30^+^ NK, PD‐L1^+^ MDSC	TNBC (*n* = 45)	αPD‐1	**↑** Baseline		**↑** (*p* < 0.001)		102
			αPD‐1 + capecitabine			**↑** (*p* < 0.01)		
Immune effector score	NK cells, CD8+PD‐1^+^ T cells, plasma sPD‐L1	NSCLC, advanced (*n* = 109)	αPD‐(L)1	**↑** Baseline	**↑** (*p* < 0.01)	**↑** (*p* < 0.001)	**↑** (*p* < 0.01)	103
Liquid immune profile‐based signature (LIPS)	CD14hi monocytes, CD8^+^ PD‐1^+^ T cells, pDCs, neutrophils, CD3^+^ CD56^+^ CD16^+^ NKTs	Solid malignancies, advanced (*n* = 89)	αPD‐(L)1	**↑** Baseline			**↑** (*p* < 0.05)	77
Logistic regression model	TGFβ, IL‐8, NLR	HPV+ malignancies (*n* = 65)	Bintrafusp alfa	**↑** Baseline			**↑** (*p* < 0.001)	85

Abbreviations: EM, effector memory; HNSCC, head and neck squamous cell carcinoma; HPV, human papillomavirus; MDSC, myeloid‐derived suppressor cells; NK, natural killer; NKT, natural killer T cells; NSCLC, non‐small‐cell lung cancer; OS, overall survival; PFS, progression‐free survival; R/M, recurrent/metastatic; RCC, renal cell carcinoma; TNBC, triple negative breast cancer; Tregs, regulatory T cells.

In 109 advanced NSCLC patients receiving αPD‐(L)1 inhibitors, patients with a high immune effector score at baseline, predicated on high levels of NK cells and CD8^+^ PD‐1^+^ T cells with low plasma sPD‐L1 concentrations, had prolonged mPFS (not reached at 20 vs. 2.3 months) and mOS (not reached at 20 vs. 4.1 months), and better response to ICI therapies than patients presenting without a high score. The predictive value of this score was improved when integrated with the Lung Immune Prognostic Index (LIPI), which is based on a derived neutrophil‐to‐lymphocyte ratio >3 along with lactate dehydrogenase levels above normal levels.^103^ The liquid immune‐profile‐based signature (LIPS), with neutrophils, CD8^+^ PD‐1^+^ T cells, pDCs, CD3^+^ CD56^+^ CD16^+^ NK T cells (NKT), and CD14^hi^ monocytes as contributors, is another proposed score to predict OS. Receiver operator characteristic (ROC) analyses identified a cutoff score of −.487. In both discovery (*n* = 56) and validation (*n* = 33) cohorts of patients with various recurrent and metastatic solid tumours treated with αPD‐(L)1, patients categorized as a low risk had improved mOS (14.5 vs. 5.4 months and 11.5 vs. 3.7 months, respectively), highlighting the tumour‐agnostic nature of this multi‐parameter model.^77^

Finally, in patients with HPV‐associated cancers (*n* = 65) treated with the bifunctional agent bintrafusp alfa, a three‐factor logistic regression model composed of baseline levels of TGFβ1, IL‐8, and the NLR associated with clinical outcome; patients having a >.5 response probability displayed a longer mOS (1061 days) than patients below this cutoff (109 days).^85^ Beyond this study, there is limited data on the predictive or prognostic utility of circulating cytokines in conjunction with one another or with other peripheral correlatives. For example, studies discussed earlier on IL‐6 and IL‐8 highlight their individual prognostic significance, though it remains unknown whether measuring these and other cytokines together would yield strengthened associations with outcomes. However, the studies discussed here underscore the utility of multi‐variate peripheral immune biomarker models to improve prediction and prognostication, and there is a need to understand how the incorporation of peripheral cytokine levels into multi‐variate models impacts their value.

## 8 FUTURE PERSPECTIVES

**FIGURE 4:**
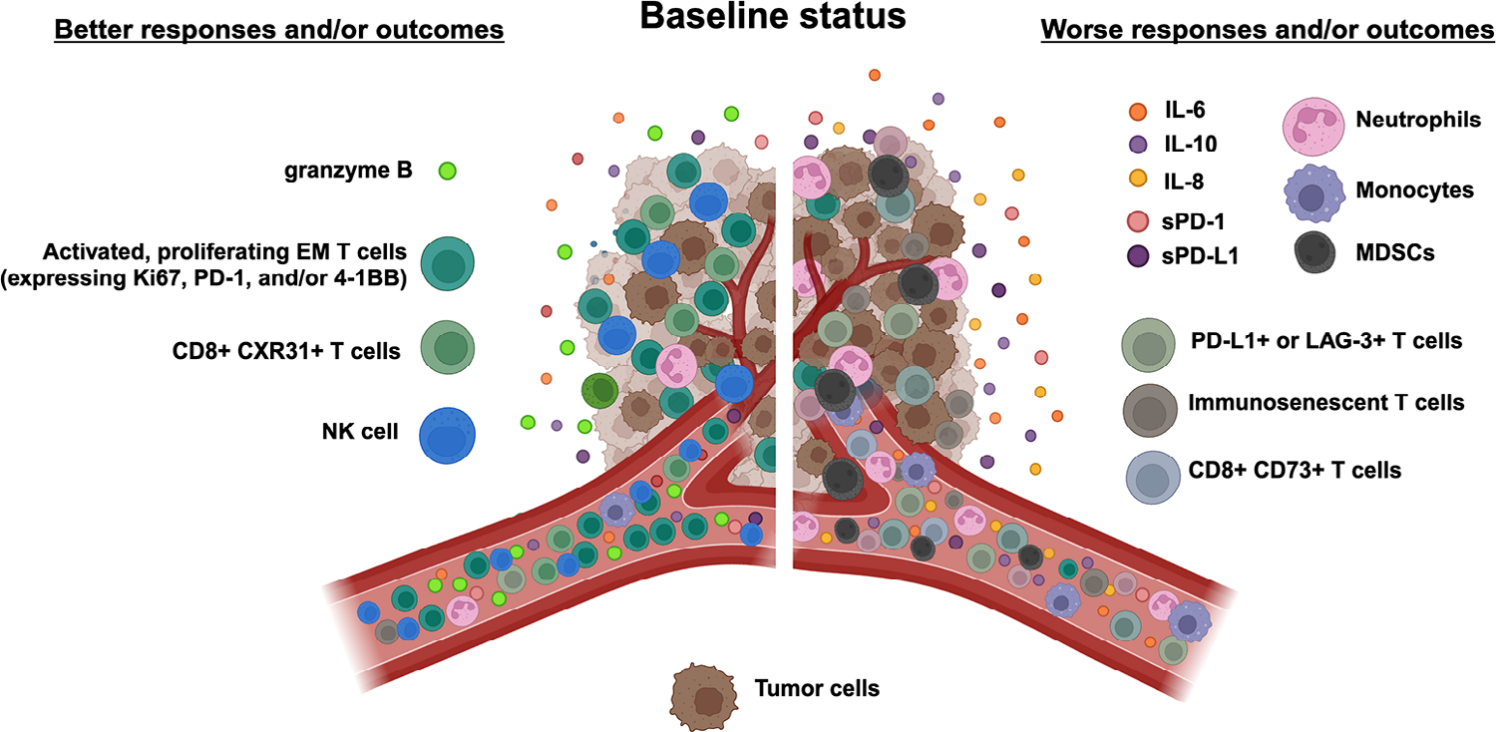
Summary of discussed candidate peripheral immune biomarkers prior to ICI therapy associated with responses and/or outcomes. EM, effector memory; MDSC, myeloid‐derived suppressor cells; NK, natural killer. Created with BioRender.com.

The peripheral immune biomarkers to guide ICI of solid tumours discussed are summarized in Figure 4. Most of the literature in this area evaluates performance characteristics of candidate biomarkers at baseline; however, some studies have evaluated how both baseline levels and changes in a given immune correlate post‐treatment compare with patient outcomes. In some cases, levels of a given biomarker at baseline associate with the response, while in others, changes in a biomarker after ICI associate with the response. There is potential for the use of peripheral immune biomarkers at baseline and post‐immunotherapy together to improve prediction and prognostication; further work is needed to determine the value of repeat sampling and optimal timepoints to interrogate a given peripheral biomarker. Of the candidates reviewed here, circulating cytokines, and IL‐8 in particular, have the most extensive body of literature supporting their use to inform response of patients to ICI therapy, both at baseline and post‐treatment. Incorporation of longitudinal cytokine assessment into laboratory‐based exploratory research of clinical trials investigating ICI is warranted. It is important to note that the data on these candidate biomarkers reviewed here have been derived from studies in multiple cancer types. Going forward, it will be essential to study whether these biomarkers have pan‐cancer utility or whether they are applicable only to particular cancer types.

Additional refined retrospective analytical methods may further biomarker discovery, such as initially comparing clinical outcomes in patients with extreme phenotypes (i.e., comparing ORR in the top and bottom 10th percentiles of the phenotypic distribution), or comparing immunological differences between patients with extreme responses. Incorporation of a basket study design into retrospective analyses across both defined and heterogeneous patient subpopulations (i.e., cancer types, same cancer but different disease stages) may enable the identification of more tumour‐agnostic biomarkers. Randomization to control for technical artifacts, batch effects, and blinding, where clinical outcomes are masked until after biomarker quantitation, can greatly diminish study biases and allow for the reproducibility of results.^104^ The proposed biomarker or model should be tested in additional cohorts with an established a priori hypothesis, preferably by multiple groups and across different centres.

The biomarkers reviewed and discussed here were not reported in a standardized manner, particularly for the peripheral immune subsets assessed by flow cytometry, where the candidate correlatives were sometimes presented as a percentage of a parent population or PBMC, or as fluorescence intensity. In addition, for some biomarkers, a high degree of variability was noted among the cutoff values that were used to stratify patients into low versus high groups for analysis. The methods used to determine cutoff values also differed among studies, with some groups utilizing the mean, median, or other percentage cutoffs, while others determined thresholds via maximally selected log‐rank statistics or by receiver operating characteristic curves. Standardized measurement and reporting are critical, very well may change the outcome of a candidate biomarker's suitability, and should be determined before widespread application. Furthermore, thorough testing of the assay platform is required to determine the accuracy, precision, and robustness of the method used to assess the biomarker or model.^105^ Another consideration here is how the timing of blood collection may impact results and whether samples procured from an individual at different times would yield the same data. Furthermore, it should be noted that all of the studies reviewed here reported on peripheral biomarkers at the population level. Prospective evaluation of a technically validated biomarker or model in a well‐annotated large study population with meaningful clinical endpoints is necessary to confirm its relevance for individual patients.^106^

It is important to note that with large prospective validation studies arise additional non‐tumour‐related considerations that may impact the peripheral biomarker development process for cancer patients receiving immunotherapy. Such factors include metabolic dysfunction, infections, chronic inflammatory diseases, and the use of medications to manage chronic conditions. Collecting data on these variables will be important for the real‐world utility of biomarkers. Controlled studies comparing the performance of peripheral biomarkers with the performance of tissue‐based biomarkers (e.g., PD‐L1 status, TMB), which are currently approved to guide treatment decisions, are extremely limited and necessary. Although identifying and validating peripheral immune biomarkers through liquid biopsy is undoubtedly complex, studies have established their foundation in guiding immunotherapy for solid tumours.

## AUTHOR CONTRIBUTIONS

Meghali Goswami, Jeffrey Schlom, and Renee N. Donahue contributed to the conception of the manuscript. Meghali Goswami, Nicole J. Toney, Stephanie C. Pitts, Carolina Celades, and Renee N. Donahue wrote the manuscript. All authors read and approved the final manuscript.

## CONFLICT OF INTEREST STATEMENT

The authors declare no conflict of interest.

## ETHICS APPROVAL AND CONSENT TO PARTICIPATE

Not applicable.

## Data Availability

Not applicable.
